# Heterotopic Ossification in a Newborn: A Case Report

**Published:** 2016-12-27

**Authors:** Khalid Murrad, Alohali Rand, Jarman Abdulaziz, Mohamed Amir Mrad

**Affiliations:** ^a^King Saud University, Riyadh, Saudi Arabia; ^b^King Faisal Specialist Hospital and Research Centre, Riyadh, Saudi Arabia

**Keywords:** heterotopic, ossification, newborn, hand, congenital

## Abstract

**Introduction:** Heterotopic ossification is defined as the formation of trabecular bone that forms outside the normal sites of the skeletal structure, materializing in soft tissue where it does not usually exist. **Methods/Case Report:** This is a case report of a 27-day-old baby with a diagnosis of DiGeorge syndrome who developed heterotopic ossification on the dorsum of his right hand. **Discussion:** Heterotopic ossification in the pediatric population is a rare finding. Very few cases were published in the literature, and we find it important to increase the knowledge on such cases and discuss possible causes with the treatment used with our patient. **Results:** General treatments of heterotopic ossification include ruling out superimposed infection, physiotherapy to prevent joint involvement, warm compressors during the active phase of development of heterotopic ossification. If the swelling persists to the point that it interferes significantly with the functional capacity of the patient or becomes a cosmetic concern, the only treatment option remaining would be surgery.

Heterotopic ossification (HO) is defined as the formation of trabecular bone that forms outside the normal sites of the skeletal structure, materializing in soft tissue where it does not usually exist.^1,2^ Although the exact pathophysiology of HO is as yet unclear, the cause can be divided into 2 types: an autosomal-dominant hereditary form known as myositis ossificans progressiva^3,4^ and an acquired form, the latter being the most common.^1^ The core concept of its development is hypothesized to be the transformation of primitive mesenchymal cells in connective tissue septa into osteogenic cells.^5^ Chalmers et al^6^ suggested that the development of HO requires the presence of 3 prerequisites: osteogenic precursor cells, inducing agents, and a conductive environment for the development of ossification. One inducing agent thought to be integral to the development of HO is the bone morphogenetic protein (BMP), as Urist et al^5^ discovered that minute amounts may be adequate to initiate the process.^5^ BMP is thought to be released from normal bone in response to venous stasis, inflammation, and diseases of connective attachments to the bone.^5^ Some investigators also suggest that prostaglandin E_2_ may have a key role in the pathogenesis of this condition through its influence on progenitor cells.^7^ HO usually develops after the incidence of trauma, spinal cord injury, or central nervous system injury.^1,8-13^ Fever, swelling, erythema, and sometimes joint tenderness are early nonspecific symptoms of HO, hence making it difficult to differentiate between cellulitis, osteomyelitis, and thrombophlebitis.^13-15^ Bone scanning and other imaging tests are then used to distinguish between these differentials. This disorder is infrequent in the pediatric population, and cases concerning newborns are but a few. In recent years, however, HO has been gaining increasing recognition, with more and more reports emerging describing its variable circumstances and presentations in infants. Thus, in this article, we present a rare case of traumatic HO in a newborn following peripheral intravenous cannulation of the right hand.

## METHODS/CASE REPORT

A 9-day-old normal spontaneous vaginal delivery full-term male infant was referred to our institute from a regional hospital as a case of chronic heart disease. After birth, he was diagnosed ultrasonically with persistent truncus arteriosus, atrial septal defect, and a perimembranous ventricular septal defect. The patient was stabilized and then discharged on anti–heart failure medications. Two days later, he was readmitted as a result of cyanosis.

On examination, the patient was found to have dysmorphic features and hypocalcemia, in addition to the congenital cardiac anomalies described earlier. Further tests were performed, and he was found to have DiGeorge syndrome, as well as cholestasis and jaundice. The patient was started on ursodiol (ursodeoxycholic acid), oral feeding with Similac 20 (Abbott, Ill), and synchronized intermittent mandatory ventilation with pressure control and support.

At the age of 27 days, the patient was found to have developed a small localized swelling on the dorsum of the right hand. It was initially thought to be an injury caused by intravenous cannulation. The mass persisted for 2 weeks and then the infant became febrile and the right hand dorsal swelling became erythematous and hot to touch. Cellulitis was suspected; thus, empiric treatment with morphine, meropenem, vancomycin, and colistin was commenced by the primary team while the septic workup was being undertaken.

Three days later, the plastic surgery service was consulted on the slowly growing swelling on the back of the hand. The plastic surgeon's examination revealed a small area of fluctuation over the mass. An ultrasound scan was ordered, and aspiration of the swelling was done and the fluid was sent for gram staining, culture, and sensitivity testing. Silver sulfadiazine cream and dressings were ordered to be applied to the wound twice daily until such time as the results of investigations were apparent. Ultrasound assessment of the hand revealed soft-tissue swelling and calcification.

On the next day, an x-ray film was obtain and the image exhibited a central area of calcification within the swelling as well. Moreover, results of blood and fluid cultures were inconclusive and found no organisms; antibiotic treatment was discontinued. However, silver sulfadiazine cream application was continued but reduced to once daily, and the hand was kept elevated. The swelling on the dorsum of the hand reduced gradually over the following few days, and dressing protocol was continued. A week later, however, at the age of 51 days, the swelling increased in size once more. Aspiration was conducted a second time, and the fluid sent for staining and culture, which proved to be negative again. Blood investigations did not yield any insight into the cause of the patient's condition either. It was at this juncture when the diagnosis of HO was considered. No further treatment was ordered except for frequent monitoring to rule out superimposed infection and cellulitis.

## DISCUSSION

We present a case report of a 27-day-old baby with a diagnosis of DiGeorge syndrome who developed HO on the dorsum of his right hand. Whether HO is related to the underlying syndrome or not is beyond the scope of this article and should involve more basic science studies to explore any relationship between DiGeorge syndrome and possible increase in BMP in the body that might trigger HO after minor trauma such as peripheral cannulation.

HO in the pediatric population is a rare finding. Very few cases were published in the literature, and we find it important to increase the knowledge on such cases and discuss possible causes with the treatment used with our patient. General treatments for HO include ruling out superimposed infection, physiotherapy to prevent joint involvement, warm compressors during the active phase of development of HO, and finally reassuring the parents that this is a benign condition with minimal effect on the patient's hand in the future besides the cosmetic appearance of the swelling.

If the swelling persist to the point that it interferes significantly with the functional capacity of the patient or becomes a cosmetic concern, the only treatment option remaining would be surgery. It is very important to ensure that HO has matured before any surgical intervention is undertaken. There are studies that show a high recurrence rate in cases that were resected before maturation.

## X-RAY FINDINGS

**Figure 1:**
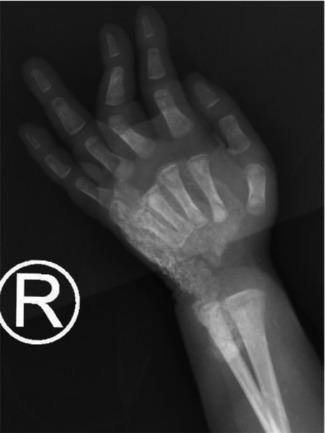
X-ray showing the dorsum of the Right hand.

**Figure 2:**
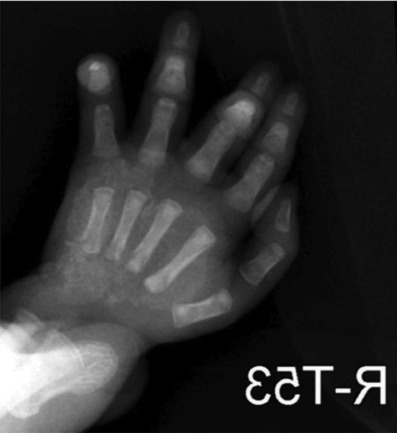
X-ray of the Right hand with medial rotation.

**Figure 3:**
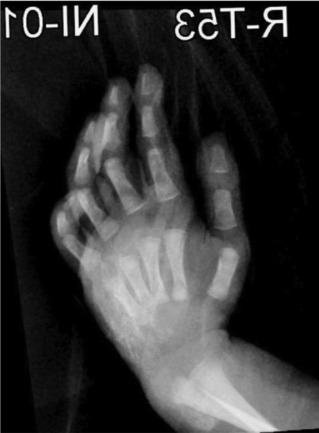
X-ray of the Right hand with flexion.

X-ray findings show diffusely increased soft-tissue thickness, swelling, and abnormal soft-tissue calcifications over the dorsal aspect of the right hand, wrist, and distal forearm with no dominant lesion. The appearance is suggestive of extensive soft-tissue calcification and swelling as shown in Figures 1, 2, and 3. Mild generalized osteopenia with bone-within-bone appearance probably related to the patient's medical or cardiac status.
